# Intratumoral heterogeneity identified at the epigenetic, genetic and transcriptional level in glioblastoma

**DOI:** 10.1038/srep22477

**Published:** 2016-03-04

**Authors:** Nicole R. Parker, Amanda L. Hudson, Peter Khong, Jonathon F. Parkinson, Trisha Dwight, Rowan J. Ikin, Ying Zhu, Zhangkai Jason Cheng, Fatemeh Vafaee, Jason Chen, Helen R. Wheeler, Viive M. Howell

**Affiliations:** 1 Sydney Neuro-Oncology Group, Bill Walsh Translational Cancer Research Laboratory, Kolling Institute, St Leonards, NSW, Australia, 2065; 2 Cancer Genetics, Hormones and Cancer Group, Kolling Institute, St Leonards, Australia, 2065; 3 Northern Sydney Local Health District, St Leonards, NSW, Australia, 2065; 4 Sydney Medical School Northern, University of Sydney, NSW, Australia, 2065; 5 Hunter New England Health, NSW, Australia, 2305; 6 Department of Physics, University of Sydney, NSW, Australia, 2006; 7 Charles Perkins Centre, University of Sydney, NSW, Australia, 2006; 8 School of Mathematics and Statistics, University of Sydney, NSW, Australia, 2006; 9 Department of Anatomical Pathology, Northern Sydney Local Health District, St Leonards, NSW, Australia, 2065.

## Abstract

Heterogeneity is a hallmark of glioblastoma with intratumoral heterogeneity contributing to variability in responses and resistance to standard treatments. Promoter methylation status of the DNA repair enzyme O^6^-methylguanine DNA methyltransferase (MGMT) is the most important clinical biomarker in glioblastoma, predicting for therapeutic response. However, it does not always correlate with response. This may be due to intratumoral heterogeneity, with a single biopsy unlikely to represent the entire lesion. Aberrations in other DNA repair mechanisms may also contribute. This study investigated intratumoral heterogeneity in multiple glioblastoma tumors with a particular focus on the DNA repair pathways. Transcriptional intratumoral heterogeneity was identified in 40% of cases with variability in *MGMT* methylation status found in 14% of cases. As well as identifying intratumoral heterogeneity at the transcriptional and epigenetic levels, targeted next generation sequencing identified between 1 and 37 unique sequence variants per specimen. *In-silico* tools were then able to identify deleterious variants in both the base excision repair and the mismatch repair pathways that may contribute to therapeutic response. As these pathways have roles in temozolomide response, these findings may confound patient management and highlight the importance of assessing multiple tumor biopsies.

Glioblastoma, a grade IV tumor of glial cell origin, is the most common and lethal primary brain tumor in adults. Tumors are genetically unstable and highly heterogeneous, displaying variable responses to therapy from patient to patient. Despite aggressive treatment involving maximal surgical resection followed by radiotherapy (RT) and concomitant and adjuvant chemotherapy (temozolomide, TMZ) median survival is just 15 months with a 5-year survival rate of less than 10%^1^. Ultimately, most patients recur as treatment resistance develops and there is no established salvage treatment that has clearly shown an improved survival benefit^2^.

Individual tumors contain a complex hierarchy of genetically distinct subclones with diverse morphologies and biological behaviors. Recent data suggest that this diversity or ‘intratumoral heterogeneity’ is one of the major reasons behind treatment failure. These diverse subclones have been shown to co-exist within the same tumor specimen^3^,^4^,^5^,^6^,^7^,^8^,^9^,^10^,^11^,^12^. It can be hypothesized that selecting therapy based on the analysis from a single biopsy specimen may not be representative of the entire lesion and could result in treatment failure. RT and alkylating chemotherapy can further exacerbate this heterogeneity by introducing additional alterations into the tumor^12^,^13^.

Such intratumoral heterogeneity undermines the usefulness of clinical biomarkers in glioblastoma and has significant implications for patient prognosis and management. Promoter methylation of the DNA repair enzyme O^6^-methylguanine DNA methyltransferase (MGMT) is the major clinically used predictive and prognostic biomarker of survival following RT and TMZ chemotherapy. Glioblastoma patients with methylated *MGMT* promoters have a more favorable outcome when treated with combination RT and TMZ versus RT alone^14^. However, a subset of patients with *MGMT* promoter methylation have poor outcome and some patients with non-methylated promoters have long survival. Intratumoral heterogeneity may be a contributing factor to this variability in response. Other cellular DNA repair mechanisms including the mismatch repair (MMR) and base excision repair (BER) pathways may also contribute and intratumoral heterogeneity in these pathways would further confound results.

The molecular subtype of the tumor can also be used as a predictive biomarker of outcome and response to therapy. Large scale molecular analysis performed by the TCGA and others has shown that glioblastoma encompasses multiple molecular subclasses of disease, each having slightly different outcomes and responses to standard of care therapy. The proneural subtype has been associated with increased survival yet no benefit was seen with aggressive therapy^15^ whereas the mesenchymal subtype has been associated with a worse outcome but a significant benefit from aggressive therapy^16^. Future stratification of patients based on molecular subtyping may improve therapy selection and outcome.

In the current study, multiple spatially distinct biopsies were taken from the same glioblastoma tumor in order to investigate intratumoral heterogeneity in key glioma biomarkers including *MGMT* promoter methylation and transcriptional subtype. We also examined whether tumors displayed intratumoral heterogeneity in the MMR and BER DNA repair pathways which may promote intrinsic or acquired treatment resistance.

## Results

### Transcriptional profiling of tumor biopsies

#### Two different limited gene panels classified tumor specimens into transcriptional subtypes with strong concordance

**Figure 1:**
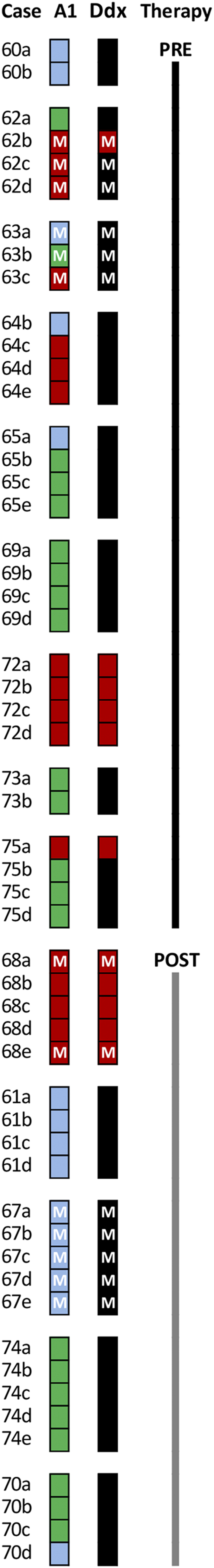
Transcriptional profiling and cluster analysis reveals intratumoral heterogeneity in 43% (6 of 14) of tumors. Intratumoral heterogeneity is defined as specimens from the same patient clustering into different groups by at least one analysis method (A1 or Ddx). Specimens were assigned to 1 of 3 groups by hierarchal clustering based on Euclidean distance of RT-qPCR results for A1 using 30 genes (SI Fig. 1, SI Table 3) and into 2 groups for Ddx by the 9 genes used for the Decision Dx panel (SI Fig. 2). “M” signifies a *MGMT* promoter methylation positive specimen. Specimens are grouped by patient and ordered by therapy. The 3 cluster groups for A1 are colored and concur with gene expression levels expected for the classical (blue), mesenchymal (green) and proneural (red) subgroups. The specimens in the red cluster group for Ddx have the highest individual scores using the Ddx algorithm suggestive of a better outcome and proneural subtype. The specimens in the black cluster group are the remaining specimens (with lower Ddx scores) and thus comprise specimens of the classical and mesenchymal subtypes.

Gene expression profiling was performed using 2 different gene panels to accurately classify the tumor biopsies into transcriptional subgroups. Genes were chosen based on previous studies as classifiers for the proneural, mesenchymal and classical transcriptional subtypes. Hierarchical clustering was performed and specimens were divided into 1 of 3 different cluster groups using the 30-gene panel (Fig. 1, A1 and SI Fig. 1). The blue group featured significantly higher mean expression of *EGFR* than either of the other groups (*p *< 0.0001, one-way ANOVA, Kruskal-Wallis test) suggestive of a classical subtype^15^. This was supported by increased mean expression of Notch (*NOTCH3, JAG1)* and Sonic Hedgehog (*GLI2)* signaling pathway genes for this group. The green group featured highest mean expression of *CHI3L1*, *CD44, SERPINE1* and *CTGF* characteristic of the mesenchymal subgroup^15^,^17^,^18^. The red group was characterized by highest mean expression for the proneural markers *DLL3, OLIG2, ASCL1* and *PDGFR*^15^,^16^,^17^,^18^,^19^.

We also performed gene expression profiling using the 9-gene Decision Dx panel (Ddx;^16^). These 9 genes were used to interrogate the cohort by both hierarchical cluster analysis and the Ddx score using ΔCt values in a weighted model for prediction of overall survival (Fig. 1, Ddx, SI Fig. 2)^16^. The top 11 ranked specimens according to the Ddx score comprised a distinct group by cluster analysis. A high Ddx score which characterized this cluster group (red group, Fig. 1, SI Fig. 2) has previously been shown to be predictive of a proneural subtype^16^.

While only 3 genes (*CHI3L1, OLIG2* and *RTN1*) were common to both the 30-gene panel and the 9-gene Ddx panel (SI Figs 1 and 2), there was strong concordance between the 2 different gene-panels for the prediction of the proneural (red) subtype with only 11% (6 of 54) of specimens discordant between A1 and Ddx. These 6 samples were ranked in the next 10 by the Ddx algorithm (SI Fig. 2) indicating different cut-off points for the different clustering algorithms with the Ddx being the most stringent for the proneural subtype.

#### Transcriptional subtyping identified intratumoral molecular heterogeneity in 40% of cases

Having classified the tumor specimens into the 3 different transcriptional sub-groups we next investigated whether the molecular heterogeneity between tumors was also evident within patients with the premise that intratumoral heterogeneity may confound the clinical utility of molecular sub-classification. Ddx identified 2 patients with intratumoral heterogeneity. In both cases, intratumoral heterogeneity was also identified by the 30-gene A1 analysis (Fig. 1). The A1 analysis further revealed heterogeneity in 40% (6 of 14) of patients. There was no significant difference in the occurrence of intratumoral heterogeneity in tumors collected pre-therapy or following recurrence (2-tailed Fisher’s Exact test). Interestingly, we did not find a significant dilution effect with differing amounts of tumour tissue. For example, specimens from the same patient which differed in tumour content by 40% were still classified as the same tumour subtype e.g. 72a (60% tumour content) and 72d (100% tumour content) and conversely, specimens taken from the same patient in which tumour content was the same (100%) were classified into different subtypes e.g. 64b and 64c. These results suggest that intratumoral heterogeneity may lead to a non-consensus transcriptional subtype in up to 40% of cases where the results are based on a single biopsy specimen.

### Intratumoral heterogeneity in *MGMT* promoter methylation status was identified in 14% of tumors and was independent of transcriptional heterogeneity

*MGMT* promoter methylation status was determined using a 4 CpG island assay that detects methylation at 4 sites in exon 1 of the human *MGMT* gene (Chr 10: 131,265,519-131,265,537), a region strongly correlated with protein expression and patient outcome^20^. *MGMT* methylation was identified in 30% (4 of 14) of cases (Table 1). Two cases were uniformly methylated across all biopsies. However, 14% (2 of 14) of cases demonstrated intratumoral heterogeneity in methylation levels with the percentage of methylation varying up to 4-fold within each case (Table 1, SI Table 4). Of note, methylation status appeared to be independent of transcriptional classification by either A1 or Ddx (Fig. 1). Similar to our results for transcriptional subtyping, we did not find a significant dilution effect with differing amounts of tumour tissue. Specimens from the same patient which differed in tumour content by 40% were still classified into the same methylation group e.g. 63a (100% tumour content) and 63c (60% tumour content) were classified as being methylated and conversely, specimens taken from the same patient in which tumour content was the same (100%) were classified into different methylation groups e.g. 68a (methylated) and 68b (unmethylated).

These results support previous reports of intratumoral heterogeneity for *MGMT* methylation levels and the possibility that reliance on a single biopsy specimen may not provide an accurate consensus of *MGMT* methylation for the tumor, reducing the clinical value of this biomarker.

### Intratumoral heterogeneity was detected for the expression of MMR and BER pathway genes *APEX1, PARP1, MSH2* and *PMS2*

**Figure 2:**
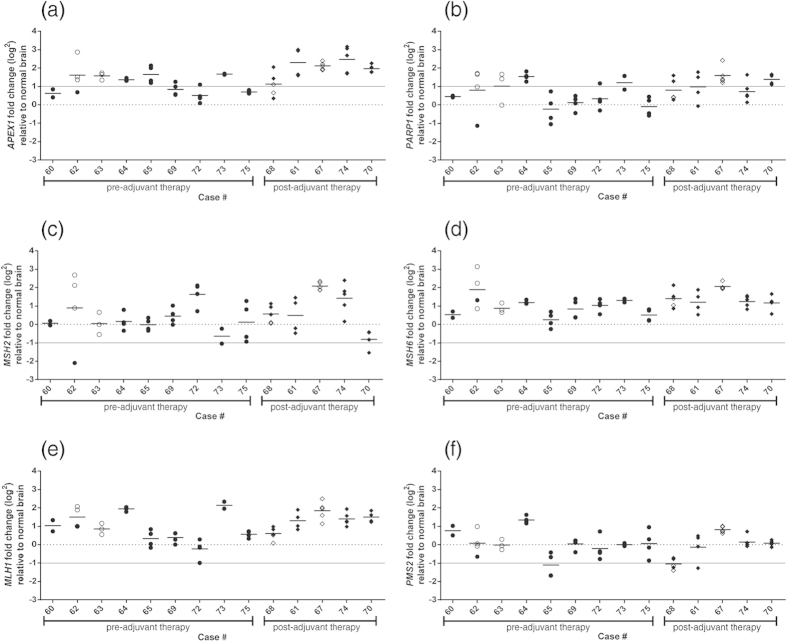
Gene expression analysis of MMR and BER pathways genes identifies both aberrant expression and intratumoral heterogeneity. The gene expression of Base Excision Repair (BER) genes (*APEX1* and *PARP1*) and Mismatch Repair (MMR) genes (*MSH6, MSH2, MLH1 and PMS2*) was examined in tumor biopsies pre- and post-adjuvant therapy (indicated by circle and diamond symbols, respectively) relative to normal brain tissue (indicated by the broken line). Results were generated using Taqman assays and *TBP* as the reference for normalization. A 2-fold change in gene expression relative to normal brain is indicated by the solid grey line. Closed symbols indicate *MGMT* promoter unmethylated samples, open symbols indicate *MGMT* promoter methylated samples.

Alterations to the MMR and BER pathways may confer resistance to TMZ independent of *MGMT* methylation. To determine whether alterations in these pathways exist in our cohort, we first assessed the gene expression levels of components of the pathways: MMR (*MSH2*, *MSH6*, *MLH1* and *PMS2*) and BER (*APEX1* and *PARP1*). Results were compared with those from normal brain (broken line, Fig. 2) and a 2-fold increase in expression for BER genes or 2-fold decrease for MMR genes set as arbitrary cut-offs for aberrant expression (solid line; 1(log_2_); Fig. 2). Intratumoral heterogeneity was defined as a greater than 2-fold range in gene expression with values spanning the cut-offs for aberrant expression.

*APEX1* was notable for being aberrantly elevated in 86% of cases in our cohort with heterogeneity of expression noted in 29% (4 of 14) of cases (Fig. 2a). *PARP1* was aberrantly elevated in at least one specimen in 71% of our cohort with intratumoral expression heterogeneity noted in 50% (7 of 14) of cases (Fig. 2b). Intratumoral heterogeneity with markedly reduced levels of the *MSH2* and *PMS2* was observed in 14% (2 of 14) and 21% (3 of 14) of cases respectively (Fig. 2c,f). No cases demonstrated intratumoral heterogeneity or markedly reduced levels of the MMR genes *MLH1* or *MSH6* (Fig. 2d,e).

There was no correlation between *MGMT* promoter methylation level and the expression of any of these genes. However, specimens from 3 of the 4 cases with methylated *MGMT* (open circles, Fig. 2) displayed aberrant levels of at least one of these genes. The intratumoral heterogeneity observed suggests the presence of clonal populations, some of which may confer decreased sensitivity to TMZ, confounding the predictive value of *MGMT* promoter methylation.

### Mutational analysis

#### Next generation sequencing of MMR and BER genes confirms intratumoral heterogeneity

Having identified the presence of inappropriate expression of members of the MMR and BER pathways we next assessed these genes at the DNA level. We performed next generation sequencing for the 6 genes of interest (*APEX1, PARP1, MSH2*, *MSH6*, *MLH1 and PMS2*) on multiple specimens from 12 cases with up to 3000x depth of coverage (average = 847x) to definitively determine whether intratumoral heterogeneity exists at this level and thus would confirm the presence of clonal or sub-clonal populations within a tumor.

**Figure 3:**
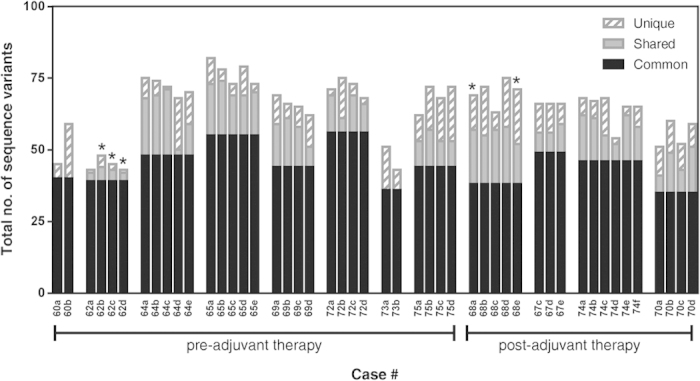
Next generation sequencing of MMR and BER genes identifies intratumoral heterogeneity at the DNA level. Targeted next generation exome sequencing results for 6 genes (*APEX1, PARP1, MSH2, MSH6, MLH2, PMS2*) performed on the Illumina Miseq platform and analyzed with Avadis NGS software. The total number of sequence variants identified for all the genes in each specimen are graphed, and specimens are grouped by case. ‘Common’ variants (those present in all specimens from a case) are illustrated in solid black bars; ‘Shared’ variants (those present in more than one but not all specimens/case) are illustrated in solid grey bars and ‘unique’ variants (those present only in one specimen/case) are illustrated in diagonal grey stripped bars. Specimens with *MGMT* promoter methylation are indicated with an asterisk (*).

After filtering for possible sequencing artifacts we identified a total of 523 sequence variants across the 6 genes in the 48 specimens sequenced from 12 cases. We found that each specimen had between 40 and 80 sequence variants in total (Fig. 3). Of these, 35 to 56 were common across each case; up to 20 were shared between at least 2 specimens of each case, and 1 to 37 variants were unique to each specimen. All specimens had sequence variants not present in the other specimens from that case, clearly demonstrating the presence of intratumoral heterogeneity.

#### Damaging DNA variants identified in DNA repair pathways

Having identified both the presence of sequence variants and intratumoral heterogeneity of these variants, we next sought to determine the functional significance of the variants identified. All variants were assessed by 2 software tools (PROVEAN and SIFT) to identify those predicted to have functional consequences. Eleven sequence variants were predicted by these software programs to be damaging and/or deleterious (SI Table 5). Four of these variants were present in sufficient abundance to be confirmed by Sanger sequencing. *PARP1 S383Y* (ENST00000366794, rs3219062), *PMS2 S46I* (ENST00000265849, rs121434629) and *APEX1 Q51H* (ENST00000216714, rs1048945) were found in all specimens from a case (with supporting reads of approximately 50% with a coverage of up to 3000x), suggestive of germline mutations. The last DNA variant confirmed by Sanger sequencing is novel and only present in an isolated specimen from a single case (specimen 68e; *PMS2 P573fs,* ENST00000265849 with supporting reads of 50%) confirming the presence of intratumoral heterogeneity.

#### The level of sequence heterogeneity varies between clinical groupings

**Figure 4:**
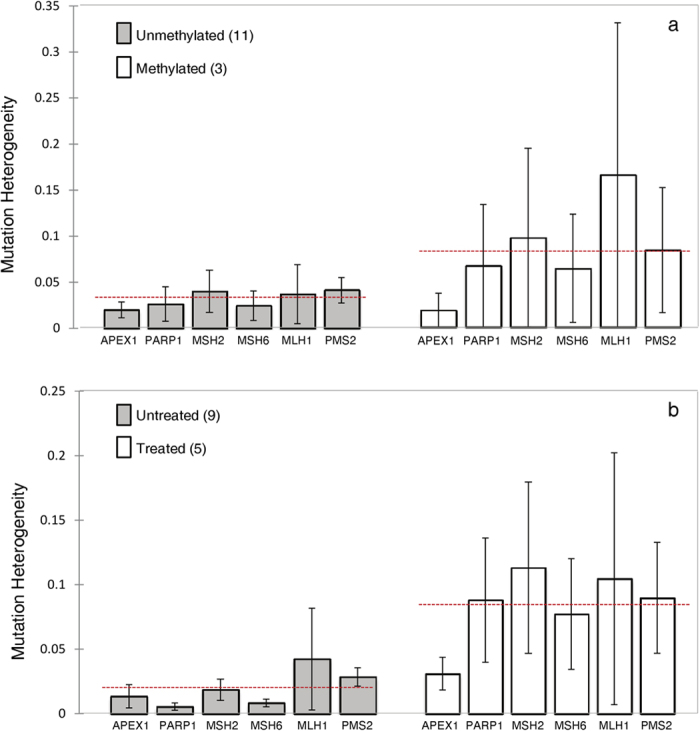
Sequence heterogeneity of BER and MMR genes. Sequence heterogeneity was calculated for each BER (*APEX1* and *PARP1*) and MMR (*MSH6, MSH2, MLH1 and PMS2*) gene for each biopsy as described in the methods. Samples were then stratified by *MGMT* promoter methylation (**a**) or treatment status (**b**). The red dotted line indicates the mean sequence heterogeneity of the samples. For cases in which *MGMT* promoter methylation heterogeneity was identified, case 62 was included in the methylated group with biopsy 62a being excluded from the analysis (as this single biopsy was determined to be unmethylated), and case 68 was included in the unmethylated group with biopsies 68a and 68e also being excluded (as they were determined to be methylated).

We next determined the level of sequence heterogeneity for each gene across clinically relevant groupings of our cohort: *MGMT* promoter methylated *versus* unmethylated and pre and post-therapy. *MGMT* promoter methylation hinders DNA repair implying that such specimens may have increased sequence heterogeneity relative to unmethylated specimens. Our results support this with sequence heterogeneity across all genes found to be 2-fold higher in *MGMT* promoter methylated *versus* unmethylated cases (Fig. 4a).

Standard of care treatment acts by damaging DNA, thereby triggering apoptosis of cells with damaged DNA. However, in the presence of resistance mechanisms apoptosis may not be triggered leading to accumulation of mutations or alternatively, the repair of the DNA damage or both in combination resulting in increased sequence heterogeneity. Comparison of post-therapy *versus* pre-therapy cases found that as anticipated there was 4-fold higher heterogeneity in post-therapy cases relative to pre-therapy cases (Fig. 4b).

## Discussion

Intratumoral heterogeneity appears to be responsible, at least in part, for resistance to standard treatments and the variable responses seen to treatment^8^,^9^,^21^. In this study we identified intratumoral heterogeneity at the transcriptional, methylation and mutational levels in multiple spatially distinct biopsies taken from up to 14 different glioblastoma cases.

Molecular classification of tumors has identified different subtypes which may be important in treatment stratification response^15^,^18^. However, a single biopsy specimen is unlikely to represent the molecular landscape of the entire tumor. Using gene clustering techniques, we observed that biopsies taken from different locations within the same tumor were classified into different molecular subtypes, in agreement with previous research^10^. Such results not only highlight the intratumoral heterogeneity that exists but also the importance of taking multiple tumor biopsies and genomically analyzing them in order to correctly classify tumors.

The most important clinical biomarker used for glioblastoma is *MGMT* promoter methylation status. Similar to our results for molecularly classifying the tumors, intratumoral heterogeneity in *MGMT* status was identified in our cohort. These findings reiterate that basing clinical management on the findings of a single biopsy specimen and a single clinical biomarker such as *MGMT* may have devastating consequences for the patients. Intratumoral heterogeneity in *MGMT* promoter methylation status has been previously demonstrated in some^22^ but not in all studies^23^. In addition, area-specific intratumoral heterogeneity for MGMT protein expression has also been identified^24^,^25^. Similar patterns of non-uniform expression have been shown with other markers^26^,^27^,^28^,^29^,^30^,^31^ demonstrating that this is not a unique feature of MGMT expression. Spatial heterogeneity has also been demonstrated by comparative genome hybridization analysis which revealed different chromosomal aberrations in different areas of the same individual tumor^10^,^31^. Thus the classification of tumors based on a single biopsy specimen can be misleading for patient stratification and patient management. However, it must be remembered that certain changes such as mutations of *IDH1* are considered early events in tumor development^12^ and are consequently found ubiquitously in tumor tissue. Distinguishing additional early driver mutations from late changes may better assist in patient stratification for treatment selection.

*MGMT* promoter methylation status is not always predictive of response to therapy. We have demonstrated intratumoral heterogeneity for *MGMT* and thus reliance on a single biopsy specimen for analysis may be misleading. However, previous studies have also demonstrated that other DNA repair mechanisms are involved. Aberrant expression of MMR and BER may confer decreased sensitivity to TMZ thus confounding the predictive value of *MGMT* methylation status. Up-regulation of BER components^32^,^33^ or loss of MMR components^34^ has been shown to promote resistance to treatment. In addition, in *MGMT* methylated patients, treatment with the alkylating agent TMZ has been shown to induce a hypermutated phenotype, resulting in DNA repair deficiency and enhanced chemo-resistance^12^,^35^,^36^,^37^,^38^. More recently, *MSH6* mutations independent of *MGMT* methylation status, have been used as biomarkers for identifying patients intrinsically deficient in DNA repair capacity making them more resistant to chemotherapy^39^. Similarly, in our cohort, predicted deleterious DNA variants were identified in both the BER and MMR pathways independent of *MGMT* methylation status. The consequences of such variants may lead to inactivation of these repair pathways and failure of treatment.

While personalized medicine for glioblastoma patients may still be in its infancy, the layers of complexity that are added due to intratumoral heterogeneity are staggering. Modulation of the DNA repair pathways may be of benefit in patients^32^,^40^,^41^,^42^,^43^ however, correctly stratifying patients for these molecular targeted treatments needs to be carefully considered. Methods which can identify phylogenetic clonality and common driver mutations would greatly enhance the ability to select appropriate treatments for these individual patients. Current practice is to take a single biopsy specimen for both diagnosis and molecular testing for *MGMT* promoter methylation or other parameters. However, our results suggest that taking multiple biopsy specimens would be more representative of the entire tumor landscape and may uncover particular aberrations which may be targetable by newly developed treatments.

Taken together our results demonstrate intratumoral heterogeneity at the epigenetic level through *MGMT* status, the gene expression level, and the DNA mutational level through sequence variation. The identified intratumoral heterogeneity highlights the importance of taking multiple biopsies from the same tumor in order to correctly classify patients. This intratumoral heterogeneity may be the reason for variations in treatment response and thus should be considered thoroughly when analyzing tumors to stratify patients. Caution must be taken when interpreting the results of a genomic biomarker from a single biopsy specimen.

## Methods

### Clinical Characteristics

Glioblastoma samples were from patients diagnosed and treated at Royal North Shore (RNS) and North Shore Private Hospitals between August 2012 and July 2013. All cases were consented and approved by Northern Sydney Local Health District Human Research Ethics Committee, under protocols 0211-171M and 1306-212M. All procedures were performed in accordance with the approved guidelines and regulations. Informed consent was obtained from all subjects. Tumor histology was reviewed by neuropathologist (J.C.) and a diagnosis of glioblastoma confirmed. Clinical data collected included age at diagnosis, gender, treatment and survival, with a minimum of 15 months follow-up on all cases (Supplementary Information [SI] Table 1). Adjuvant therapy included TMZ and RT or RT alone.

### Fresh Frozen Tumor Tissue

A cohort of 14 glioblastoma cases was available. For each case, up to six tumor tissue biopsies (5–10 mm^3^) were harvested at surgery, from regions at least 1cm apart. Tissue was immediately snap frozen and stored at −80 °C until further analysis.

### Tumor Tissue Quality Control

In order to assess tumor tissue quality a small section of each biopsy (3–5 mm^3^) was cut, fixed in formalin, and stained with haematoxylin and eosin (H&E) for scoring by neuropathologist (J.C.). Slides were scored for percentage volume of tumor, necrosis and non-tumor tissue.

### Extraction of DNA and RNA

DNA and RNA was extracted from all remaining tissue from each biopsy using the Allprep DNA/RNA/Protein kit (Qiagen, Valencia, CA) as per manufacturer’s instructions. For all samples, purity was assessed using a Nanodrop ND-1000 (Thermo Scientific, Wilmington, DE) and for deep sequencing, DNA samples were further quantified using the Qubit dsDNA HS Assay Kit (Q32851, Life Technologies, Mulgrave, Victoria), performed on the Qubit Fluorometer 1.0 (Life Technologies).

### Analysis of IDH1 mutation status

*IDH1* mutation status was determined by Sanger sequencing of exon 4. DNA was amplified by polymerase chain reaction (PCR), using primers spanning exon 4 (5′-CATTTGTCTGAAAAACTTTGCTT-3′ (forward) and 5′-TCACATTATTGCCAACATGAC-3′ (reverse); amplicon size: 359 bp). PCR products were purified using the DNA Clean and Concentrator Kit (Zymo Research, Irvine, CA), and commercially sequenced (Australian Genome Research Facility, Westmead, Australia). Immunohistochemical screening for *IDH1* mutation-positive cases was also performed by Pathology North, RNS Hospital, using the monoclonal antibody against IDH1 p.R132H (Clone H09; Dianova, Germany).

### Determination of MGMT Promoter Methylation Status

DNA extracted from frozen tumor tissue was tested for *MGMT* promoter methylation by commercial pyrosequencing (University of Sydney). The assay threshold was determined by averaging the percent methylation at all 4 CpG sites in 4 non-neoplastic brain tissue samples (previously confirmed as being unmethylated by both pyrosequencing and methylation-specific PCR), and applying 2 standard deviations as previously reported by Dunn *et al*.^44^. Control samples were analyzed in 3 to 5 independent pyrosequencing runs, giving a mean of 5.49% (SD 3.85) and a positive methylation assay threshold of 13%.

### Gene Expression Profiling

Following quality analysis and quantification, 1 μg RNA was treated with DNase 1, amplification grade (Life Technologies) and reverse transcribed using the SuperScript III First Strand Synthesis SuperMix Kit (Life Technologies) according to the manufacturer’s instructions. Gene expression was then analyzed commercially using the Fluidigm 96.96 BioMark HD System (Ramaciotti Gene Analysis Centre, Randwick, NSW, Australia) as per manufacturer’s instructions. The NormFinder algorithm^45^ was used to compare 5 endogenous control genes included on the array (*TBP, ACTB, GAPDH, IPO8* and *SDHA*), identifying *TBP* as being the most stable control gene. Relative expression of target genes was determined using the 2^−delta-delta Ct^ method (Fluidigm Real-time PCR analysis software), normalizing expression to *TBP* and a commercial pooled normal brain control sample (calibrator; Ambion, Life Technologies).

Genes of interest were then validated in triplicate using the Applied Biosystems 7900HT real time PCR system and individual TaqMan assays following the manufacturer’s instructions. Results were analyzed using Data Assist Software v3.0 (Life Technologies) and fold changes in each individual gene expression were calculated independently using the 2^−delta-delta Ct^ method after normalizing to *TBP.*

### Classification of Transcriptional Subtypes

Ct values from the Fluidigm and AB7900HT system (*LGALS3/TBP* only) were imported into the HTqPCR package in Bioconductor^46^ and unreliable data filtered out by applying a Ct cut off value of 40. Genes with errors detected in less than 1% of samples were retained in the analysis by imputing median values for those samples. Samples with Ct values greater than 40 for *CCND2* were given a Ct = 40 to retain these in the analysis. *TBP* was used for delta Ct normalization. The Euclidean distance metric was used for hierarchical clustering of samples into transcriptional subclasses using a panel of genes previously found to be differentially expressed between subtypes^47^ or the DecisionDx-GBM®^16^ gene expression panel.

### Targeted Next Generation Sequencing

An amplicon library was created using 250 ng of genomic DNA from tumor specimens as per the manufacturer’s instructions (TruSeq Custom Amplicon Library Kit, Illumina, Scoresby, Victoria). A custom oligo panel was designed using Illumina Design Studio software and the UCSC genome browser to improve coverage, avoiding regions of low complexity sequence and CpG rich sequence. Primers were designed against all exonic regions of the six MMR/BER genes of interest (*APEX1, PARP1, MSH2, MSH6, MLH1* and *PMS2*) using a total of 183 amplicons, representing 99% of the coding sequence. The exome library was then pooled and sequenced on the Illumina MiSeq platform as 250-bp paired end reads (MiSeq Reagent Kit v2, 300 cycles) according to the Illumina protocol. Primary analysis of deep sequencing data was performed by the MiSeq Illumina reporter software (using default settings), including alignment of sequence reads to the human genome reference GRCh37 (hg19).

### DNA Variant Identification

Sequenced reads were mapped to the hg19 reference human genome (Ensembl Gene annotation 2014.01.02) using Avadis NGS v1.6 software (Strand Life Sciences, India). After filtering out low-quality and duplicate reads, single nucleotide variants (SNVs) and short insertions/deletions were identified using pipeline analysis in the Avadis software and default criteria (≥20X coverage, an average base quality ≥20 with a confidence score cutoff greater than 50, ≥10 reads coverage of the specific variant location and ≥2 reads of the variant).

### Identification of Predicted Significant variants

PROVEAN (Protein Variant Effect Analyzer) online software tool v1.1.3^48^ was used to predict which variants may have damaging or deleterious effects according to PROVEAN and SIFT (Scale-Invariant Feature Transform) predictions.

### Sanger sequencing

To validate the DNA variants predicted to be damaging or deleterious, Sanger sequencing was performed. Primers were designed using Primer 3 (v.0.4.0) to amplify ~200 bp amplicon containing the region of interest (SI Table 5). PCR reactions were carried out in a total of 30 μL which included ~100 ng genomic DNA, 6 μL HOT FIREPOL blend mix (Solis BioDyne, Tartu, Estonia) and 0.5 uM primers with cycling conditions (95 °C for 14 mins; 28 cycles of 95 °C for 15 secs, 58 °C for 30 secs, 72 °C for 1 min; 72 °C for 7 mins). Amplicons were purified and commercially sequenced as described above.

### Sequence heterogeneity calculation

The sequence variants were used to calculate the sequence heterogeneity between the different biopsy specimens from each patient using MATLAB® R2013a (Mathworks, MA). Each gene was analysed individually. The total number of DNA variants in all biopsies for each gene was first identified (i.e. the total number of all DNA variants identified from all biopsies combined together). The number of DNA variants for each individual biopsy was then compared to this number to generate a ‘sequence variant percentage’ for each biopsy or, the number of DNA variants in an individual biopsy divided by the total number of DNA variants identified overall. This sequence variant percentage was then used to compare individual biopsy from the same patient together with similar percentages implying low heterogeneity. Samples were then stratified according to *MGMT* promoter methylation or treatment status.

## Additional Information

**How to cite this article**: Parker, N. R. *et al*. Intratumoral heterogeneity identified at the epigenetic, genetic and transcriptional level in glioblastoma. *Sci. Rep.* **6**, 22477; doi: 10.1038/srep22477 (2016).

## Supplementary Material

Supplementary Information

## Figures and Tables

**Table 1:** Cohort details including *MGMT* promoter methylation status that demonstrates intratumoral heterogeneity (M: Methylated promoter, U: Unmethylated promoter, RT: radiotherapy, TMZ: temozolomide chemotherapy).

Case #	Surgery	Treatment	**MGMT promoter methylation status (M: > 13%, U: < 13%)**					
			**Biopsy A**	**Biopsy B**	**Biopsy C**	**Biopsy D**	**Biopsy E**	**Biopsy F**
**60**	1	Naïve	U	U				
**62**	**1**	**Naïve**	**U (4.3)**	**M (20.3)**	**M (32.8)**	**M (16.3)**		
**63**	1	Naïve	M	M	M			
**64**	1	Naïve	U	U	U	U	U	
**65**	1	Naïve	U	U	U	U	U	
**69**	1	Naïve	U	U	U	U		
**72**	1	Naïve	U	U	U	U		
**73**	1	Naïve	U	U				
**75**	1	Naïve	U	U	U	U		
68*	**2**	**RT**	**M (33.0)**	**U (9.8)**	**U (8.8)**	**U (11.8)**	**M (16.5)**	
**61**	2	RT-TMZ	U	U	U	U		
**67**	2	RT-TMZ	M	M	M	M	M	
**74**	2	RT-TMZ	U	U	U	U	U	U
**70**	3	RT-TMZ	U	U	U	U		

For cases where variability was detected, the percentage methylation is shown in brackets.

^*^IDH1 mutation identified; secondary glioblastoma.
