# Supplementary material for: Intratumoral heterogeneity identified at the epigenetic, genetic and transcriptional level in glioblastoma
Source: Sci Rep. 2016 Mar 4;6:22477. doi: 10.1038/srep22477 (PMC4778014; doi:10.1038/srep22477)
Supplement: Supplementary Information [file srep22477-s1.doc]

**Supplementary Data**

Title: Intratumoral heterogeneity identified at the epigenetic, genetic and transcriptional level in glioblastoma

Nicole R. Parker, Amanda L. Hudson, Peter Khong, Jonathon F. Parkinson, Trisha Dwight, Rowan J. Ikin, Ying Zhu, Zhangkai Jason Cheng, Fatemeh Vafaee, Jason Chen, Helen R. Wheeler, Viive M. Howell.

**SI Table 1: Characteristics of the Study Cohort (n=14 glioblastoma cases).**

**Supplementary Table 1:** Patient cohort clinical data outlining age at diagnosis and overall survival (OS) calculated from date of diagnosis with a glioblastoma till date of death.

| **Case #** | **Sex** | **Age at Diagnosis (Years)** | **OS since diagnosed with a glioblastoma (months)** |
| --- | --- | --- | --- |
| **60** | M | 81.3 | 28.46 |
| **62** | F | 56.7 | 19.0 |
| **63** | M | 81.9 | 18.89 |
| **64** | M | 46.9 | 13.26 |
| **65** | M | 75.9 | 12.5 |
| **69** | M | 78.5 | 29.1 |
| **72** | F | 79.2 | 2.3 |
| **73** | M | 63.2 | 17.0 |
| **75** | F | 63.9 | 25.7 |
| **68*** | M | 47.1 | 3.5 |
| **61** | M | 81.3 | 13.2 |
| **67** | M | 50.4 | 43.9 |
| **74** | F | 48.7 | 10.9 |
| **70** | M | 40.9 | 47.9 |
|  | **Mean:** | **64.0** | **20.4** |
|  | **Median:** | **63.6** | **17.9** |

**IDH1* mutation identified; secondary glioblastoma.

**SI Table 2: *MGMT* promoter methylation results for determining the assay cut off values.** The cut-off value was determined by averaging the percentage methylation at 4 CpG sites of known unmethylated controls and applying 2 standard deviations. Samples were considered methylated if the average percentage methylation was >13% or unmethylated if the average percentage methylation was <13%. FFPE, formalin fix paraffin embedded.

| **Sample** | **Average % methylation** |
| --- | --- |
| Normal brain FFPE tissue (n=13) | 9.8 |
|  | 1 |
|  | 4.8 |
|  | 4.8 |
|  | 3.5 |
|  | 1.8 |
|  | 2.3 |
|  | 9.5 |
|  | 15.3 |
|  | 11.8 |
|  | 6.5 |
|  | 6 |
|  | 12.5 |
| Normal brain fresh/frozen tissue (n=3) | 4.3 |
|  | 3.8 |
|  | 3.5 |
| Unmethylated control (n=8) | 2 |
|  | 9 |
|  | 5.3 |
|  | 4.8 |
|  | 2.8 |
|  | 2.3 |
|  | 2.5 |
|  | 2 |
|  |  |
| **Mean percentage methylation** | **5.49** |
| **Standard deviation** | **3.85** |
| **Assay cut off Mean + 2 standard deviations** | **13%** |

**SI Table 3: TaqMan assays used for gene expression profiling.**

| **Gene symbol** | **Taqman Probe ID** |
| --- | --- |
| AQP1 | Hs01028916_m1 |
| ASCL1 | Hs04187546_g1 |
| ATRX | Hs00230877_m1 |
| C/EBPBeta | Hs00270923_s1 |
| CD44 | Hs01075861_m1 |
| CDKN2A | Hs00923894_m1 |
| CTGF | Hs01026927_g1 |
| DCX | Hs00167057_m1 |
| DLL1 | Hs00194509_m1 |
| DLL3 | Hs01085096_m1 |
| EGFR | Hs01076078_m1 |
| EMP3 | Hs00171319_m1 |
| FN1 | Hs00365052_m1 |
| GAPDH | Hs00183533_m1 |
| GLI2 | Hs01119974_m1 |
| GPNMB | Hs01095679_m1 |
| HES1 | Hs00172878_m1 |
| HMBS | Hs00609293_g1 |
| IGFBP2 | Hs01040719_m1 |
| IPO8 | Hs00183533_m1 |
| JAG1 | Hs01070032_m1 |
| LGALS3^*^ | Hs00173587_m1 |
| NBN/NBS1 | Hs01039836_m1 |
| NCAM1 | Hs00941830_m1 |
| NCAM2 | Hs00189850_m1 |
| NF1 | Hs01035108_m1 |
| NOTCH1 | Hs01062014_m1 |
| NOTCH3 | Hs01128541_m1 |
| OLIG2 | Hs00300164_s1 |
| OMG | Hs03047013_s1 |
| PDGFRA | Hs00998018_m1 |
| PDPN | Hs00366766_m1 |
| RTN1 | Hs00382515_m1 |
| SDHA | Hs00188166_m1 |
| Serpine1 | Hs01126607_g1 |
| SOX 9 | Hs01001343_g1 |
| STAT3 | Hs00374280_m1 |
| TBP | Hs00427620_m1 |
| TNFalpha | Hs01113624_g1 |
| VEGFA | Hs00900055_m1 |
| YKL40/CHI3L1 | Hs00609691_m1 |

*Data from the AB7900HT platform only.

**SI Table 4: MGMT promoter methylation results for each tumour specimen.**

Average percentage methylation was determined at 4 CpG sites using pyrosequencing. M, methylated MGMT promoter; U, unmethylated MGMT promoter.

| **Case** | **CpG1%** | **CpG2%** | **CpG3%** | **CpG4%** | **Average % methylation** | **Methylation status (M:>13%, U:<13%)** |
| --- | --- | --- | --- | --- | --- | --- |
| **60a** | 1 | 1 | 3 | 3 | 2 | unmethylated |
| **60b** | 2 | 3 | 8 | 7 | 5 | unmethylated |
| **62a** | 3 | 5 | 3 | 6 | 4.25 | unmethylated |
| **62b** | 33 | 13 | 8 | 27 | **20.25** | **methylated** |
| **62c** | 48 | 14 | 11 | 58 | **32.75** | **methylated** |
| **62d** | 17 | 17 | 10 | 21 | **16.25** | **methylated** |
| **63a** | 60 | 61 | 64 | 66 | **62.75** | **methylated** |
| **63b** | 39 | 44 | 48 | 53 | **46** | **methylated** |
| **63c** | 47 | 53 | 51 | 62 | **53.25** | **methylated** |
| **64a** | 2 | 2 | 5 | 5 | 3.5 | unmethylated |
| **64b** | 3 | 3 | 6 | 6 | 4.5 | unmethylated |
| **64c** | 0 | 1 | 3 | 3 | 1.75 | unmethylated |
| **64d** | 2 | 2 | 6 | 6 | 4 | unmethylated |
| **64e** | 2 | 1 | 5 | 5 | 3.25 | unmethylated |
| **65a** | 3 | 3 | 7 | 7 | 5 | unmethylated |
| **65b** | 6 | 7 | 4 | 6 | 5.75 | unmethylated |
| **65c** | 3 | 4 | 5 | 7 | 4.75 | unmethylated |
| **65d** | 8 | 11 | 7 | 11 | 9.25 | unmethylated |
| **65e** | 8 | 8 | 8 | 11 | 8.75 | unmethylated |
| **69a** | 1 | 1 | 2 | 3 | 1.75 | unmethylated |
| **69b** | 3 | 7 | 2 | 14 | 6.5 | unmethylated |
| **69c** | 2 | 3 | 3 | 14 | 5.5 | unmethylated |
| **69d** | 1 | 1 | 2 | 3 | 1.75 | unmethylated |
| **72a** | 6 | 12 | 7 | 10 | 8.75 | unmethylated |
| **72b** | 9 | 10 | 7 | 17 | 10.75 | unmethylated |
| **72c** | 5 | 9 | 7 | 12 | 8.25 | unmethylated |
| **72d** | 9 | 7 | 6 | 17 | 9.75 | unmethylated |
| **73a** | 2 | 2 | 4 | 5 | 3.25 | unmethylated |
| **73b** | 2 | 3 | 5 | 7 | 4.25 | unmethylated |
| **75a** | 1 | 2 | 3 | 3 | 2.25 | unmethylated |
| **75b** | 1 | 1 | 2 | 3 | 1.75 | unmethylated |
| **75c** | 2 | 2 | 5 | 6 | 3.75 | unmethylated |
| **75d** | 1 | 2 | 2 | 3 | 2 | unmethylated |
| **68a** | 30 | 38 | 24 | 40 | **33** | **methylated** |
| **68b** | 5 | 14 | 6 | 14 | 9.75 | unmethylated |
| **68c** | 3 | 5 | 4 | 23 | 8.75 | unmethylated |
| **68d** | 8 | 9 | 6 | 24 | 11.75 | unmethylated |
| **68e** | 9 | 16 | 13 | 28 | **16.5** | **methylated** |
| **61a** | 1 | 1 | 2 | 4 | 2 | unmethylated |
| **61b** | 2 | 2 | 2 | 4 | 2.5 | unmethylated |
| **61c** | 1 | 1 | 2 | 3 | 1.75 | unmethylated |
| **61d** | 1 | 1 | 2 | 3 | 1.75 | unmethylated |
| **67a** | 40 | 45 | 15 | 48 | **37** | **methylated** |
| **67b** | 54 | 58 | 11 | 63 | **46.5** | **methylated** |
| **67c** | 55 | 62 | 10 | 66 | **48.25** | **methylated** |
| **67d** | 9 | 50 | 5 | 49 | **28.25** | **methylated** |
| **67e** | 6 | 34 | 7 | 17 | **16** | **methylated** |
| **74a** | 3 | 3 | 6 | 6 | 4.5 | unmethylated |
| **74b** | 0 | 0 | 2 | 3 | 1.25 | unmethylated |
| **74c** | 1 | 2 | 3 | 4 | 2.5 | unmethylated |
| **74d** | 1 | 1 | 2 | 3 | 1.75 | unmethylated |
| **74e** | 2 | 2 | 3 | 5 | 3 | unmethylated |
| **74f** | 1 | 2 | 4 | 5 | 3 | unmethylated |
| **70a** | 1 | 1 | 2 | 2 | 1.5 | unmethylated |
| **70b** | 1 | 1 | 2 | 2 | 1.5 | unmethylated |
| **70c** | 2 | 1 | 4 | 4 | 2.75 | unmethylated |
| **70d** | 3 | 4 | 5 | 3 | 3.75 | unmethylated |

**SI Table 5: Primers and amplicon size for Sanger sequencing of predicted damaging DNA variants.** Fwd, forward primer; Rev, reverse primer; fs, frameshift.

| DNA variant | Primer 5’-3’ | Amplicon length (base pairs) |
| --- | --- | --- |
| PARP1 L641fs | Fwd - TACGGTGATCGGTAGCAACA  Rev - AAGGTGTCCCTTCCTTTTCC | 216 bp |
| PARP1 S383Y | Fwd - CCAGAAACCAGCGCCTCC  Rev - TGGGGTCAGCAAAGAGAGAC | 205 bp |
| MSH6 F432fs | Fwd - GAGATGAGCACAGGAGGAGG  Rev - GCCCAGTTGCCTTTCATGAA | 235 bp |
| MSH6 T986FS | Fwd - CCTAGAGAAACAGCGCAACA  Rev - TCCCTCCGTTCTTCAGCATT | 216 bp |
| PMS2 D69E | Fwd - ACTGATAGCATGGGTCCGTT  Rev - TGCATTTCCCAAGACAGTGT | 225 bp |
| PMS2 H832fs | Fwd - GAAGAGCTGCCATTCTGACG  Rev - AGGTTGGCGATGTGTCTCA | 186 bp |
| PMS2 S45I | Fwd - GTGTTGAGTCATTTCCCACAGT  Rev - TGGCTTAAAACTCTCCCAAACT | 250 bp |
| PMS2 P573fs | Fwd - GGACTGCCATTCAAACCAGG  Rev - GAGGCTGACATGTCCTGAGT | 162 bp |
| APEX1 F252fs | Fwd - AAAAGAATGCTGGCTTCACG  Rev - CTTGGAACGGATCTTGCTGT | 230 bp |
| APEX1 Q50H | Fwd - AAGAAGTCGCAGGAACCGTA  Rev - TCTTGAGTGTGGCAGGTTTG | 235 bp |
| MLH1 L352F | Fwd - CTCCATTTGGGGACCTGTAT  Rev - CATCTGGTGGGCATAGACCT | 213 bp |

**SI Figure 1: Heatmap and dendrogram of hierarchical clustering of the 54 specimens in our cohort using the 30-gene panel for A1.**

**
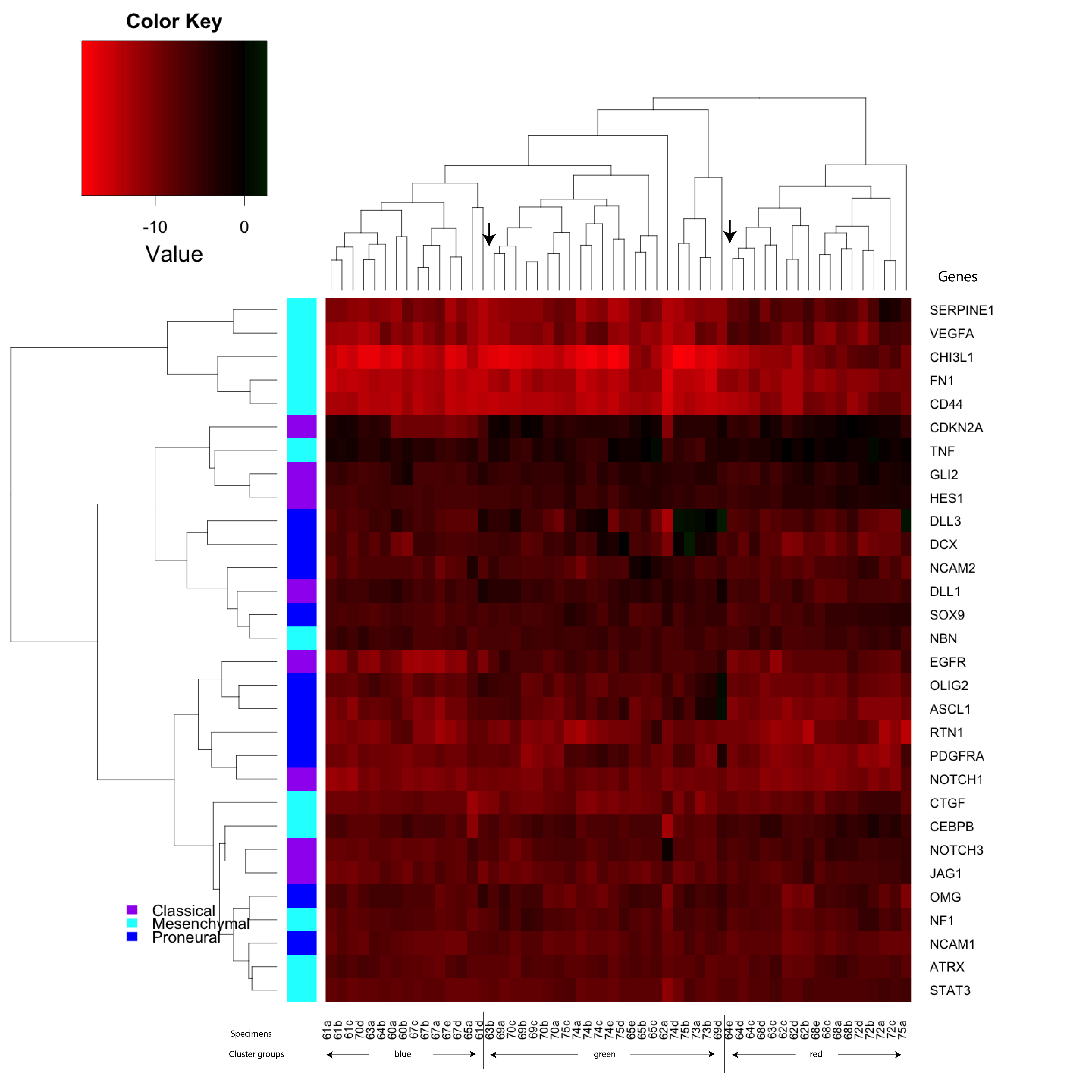
**

**SI Figure 2: Heatmap and dendrogram of hierarchical clustering of the 54 specimens in our cohort using the 9 Ddx genes. Ddx ranking by score is also listed.**

**
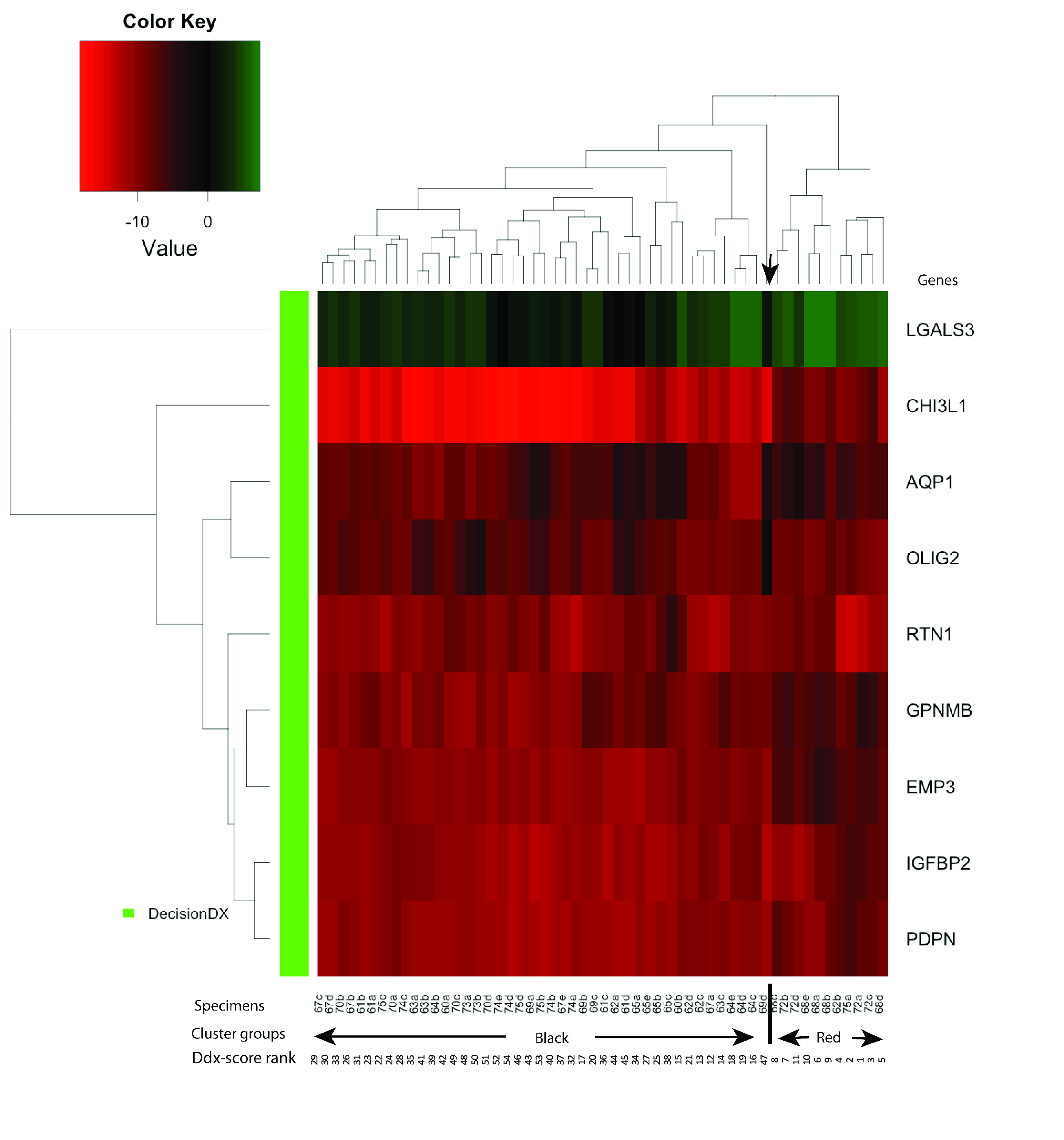
**
